# Implication of SPARC in the modulation of the extracellular matrix and mitochondrial function in muscle cells

**DOI:** 10.1371/journal.pone.0192714

**Published:** 2018-02-08

**Authors:** Aicha Melouane, Antoine Carbonell, Mayumi Yoshioka, Jack Puymirat, Jonny St-Amand

**Affiliations:** 1 CREMI, CHU de Québec Research Center, Quebec, Quebec, Canada; 2 Department of Molecular Medicine, Faculty of Medicine, Laval University, Quebec, Quebec, Canada; 3 Unit of Human Genetics, CHU de Québec Research Center, Quebec, Quebec, Canada; 4 Department of Medicine, Faculty of Medicine, Laval University, Quebec, Quebec, Canada; McGill University, CANADA

## Abstract

Secreted protein, acidic and rich in cysteine (SPARC) is differentially associated with cell proliferation and extracellular matrix (ECM) assembly. We show here the effect of exogenous SPARC inhibition/induction on ECM and mitochondrial proteins expression and on the differentiation of C2C12 cells. The cells were cultured in growth medium (GM) supplemented with different experimental conditions. The differentiation of myoblasts was studied for 5 days, the expressions of ECM and mitochondrial proteins were measured and the formation of the myotubes was quantified after exogenous induction/inhibition of SPARC. The results indicate that the addition of recombinant SPARC protein (rSPARC) in cell culture medium increased the differentiation of C2C12 myoblasts and myogenin expression during the myotube formation. However, the treatment with antibody specific for SPARC (anti-SPARC) prevented the differentiation and decreased myogenin expression. The induction of SPARC in the proliferating and differentiating C2C12 cells increased collagen 1a1 protein expression, whereas the inhibition decreased it. The effects on fibronectin protein expression were opposite. Furthermore, the addition of rSPARC in C2C12 myoblast increased the expression of mitochondrial proteins, ubiquinol-cytochrome c reductase core protein II (UQCRC2) and succinate dehydrogenase iron-sulfur subunit (SDHB), whereas the anti-SPARC decreased them. During the differentiation, only the anti-SPARC had the effects on mitochondrial proteins, NADH dehydrogenase ubiquinone 1 beta subcomplex subunit 8 (NADHB8), SDHB and cytochrome c oxidase 1 (MTCO1). Thus, SPARC plays a crucial role in the proliferation and differentiation of C2C12 and may be involved in the link between the ECM remodeling and mitochondrial function.

## Introduction

Adult mammalian skeletal muscle tissue is composed of multinucleated contractile muscle cells and it represents approximately 40% of the total body mass. The muscle fibers are surrounded by a dynamic structure named extracellular matrix (ECM) which contains collagen, glycoproteins and proteoglycans [1]. It is well known that ECM plays a crucial role in muscle cell development, structure maintenance, force transmission, and repair through the modulation of growth factors and ECM molecules interactions as well as cell-matrix signal transduction pathways [2]. Moreover, the myofibril assembly in skeletal muscle cells may be concerned by cell-matrix association. Thus, ECM modulates crucial cellular functions (adhesion, migration, proliferation and differentiation) and itself assembly by integrin-ligand combinations.

Skeletal muscle contains collagens type I and III which are fibrillar in nature. Furthermore, earlier studies have reported the importance of collagen as a substrate in the fusion of myoblasts into myotubes and showed the influence of ECM on myogenesis [3]. Multinucleated myotubes formation is an important step in skeletal muscle development. Myogenesis is a complex process characterized by the expression of myogenic regulatory factors (MRF) including myogenic factor-5 (Myf5), myoblast determination protein (MyoD), myogenin and MRF4 which led to cell division [4]. The analysis of the transcriptional changes during the differentiation of C2C12 myoblasts has shown that myogenin is an early marker for the entry of myoblasts into the differentiation pathway and that this key transcription factor governed the terminal differentiation [5]. However, not only MRF are involved in the regulation of skeletal muscle differentiation, ECM components can also play a critical role in the myogenic process [6]. Additionally, previous study has demonstrated the importance of ECM proteins in the differentiation of skeletal muscle [7]. On the other hand, ECM associated proteins, also termed matricellular proteins, do not play an architectural role in the ECM. Their interactions with cell-surface receptors, as well as with the structural matrix proteins as collagen modulate cell function and can be involved in tissue development, in satellite cell maintenance, activation, proliferation and differentiation during skeletal muscle regeneration [8, 9]. Moreover, the analysis of the skeletal muscle transcriptome after mild-exercise training in elderly has revealed the induction of 3 transcripts related to ECM, namely collagen type III alpha 1, collagen type IV alpha 1 and secreted protein, acidic and rich in cysteine (SPARC), which accounted for 25% (3/12) of modulated transcripts in elderly [10].

SPARC also known as osteonetin or basement membrane-40, is a calcium binding matricellular glycoprotein secreted by several types of cells and is associated with development, tissue remodeling, repair and injury [11]. In skeletal muscle, SPARC is expressed during muscle development and in regenerating muscle as well as in satellite cells/myoblasts and in myotubes and muscle fibers, suggesting a crucial role for SPARC in the skeletal muscle compartment [12]. SPARC is a multifunctional protein implicated in osteogenesis, wound healing, angiogenesis and disease pathogenesis [13]. Further, *in vitro* studies demonstrated that SPARC concerns cell shape modulation [14], cell cycle inhibition [15], the disruption of cell adhesion [16] and the regulation of cell differentiation [17]. Moreover, it is well known that SPARC binds to collagen type I-V and VIII [14, 18, 19], affects collagen production and assembly resulting in the modification of the ECM. For instance, collagen I deficiency deteriorates SPARC deposition in the ECM of Mov-13 mice [18] and the dermis of SPARC-null mice display a reduced collagen content [20]. Taken together, a functional relationship between SPARC and collagen I has been proposed. Furthermore, a previous study reported the interaction of SPARC with adenosine monophosphate activated protein kinase (AMPK) [21], which is known to induce the master regulator of mitochondrial biogenesis, peroxisome proliferator-activated receptor gamma coactivator 1 alpha (PGC1a) [22]. Thus, SPARC may contribute to the improvement of mitochondrial function and energy metabolism via the regulation of mitochondrial proteins expression.

Therefore, this study aimed to investigate the effect of SPARC on ECM remodeling, muscle differentiation and mitochondrial function. First, we confirmed the previous findings that the treatment with antibody specific for SPARC (anti-SPARC) prevents the differentiation of C2C12 myoblasts [23] and investigate whether the addition of recombinant SPARC protein (rSPARC) to C2C12 myoblasts promotes their differentiation using myogenin as a differentiation marker. To identify the modulator effect of SPARC on the ECM, we measured the levels of collagen 1a1 and fibronectin proteins during the proliferation and the differentiation of C2C12 cells as well as after the formation of mature myotubes. Finally, we also reported that the addition of rSPARC in C2C12 myoblast increased the mitochondrial oxidative phosphorylation (OXPHOS) proteins expression, whereas the anti-SPARC showed the opposite results during proliferation and during the differentiation of C2C12 myoblasts. These results indicate that SPARC plays a key role in the differentiation of C2C12 myoblasts and it may be involved in the link between the ECM remodeling and mitochondrial function.

## Materials and methods

http://dx.doi.org/10.17504/protocols.io.jsycnfw [PROTOCOL DOI]

### Materials

Mouse skeletal muscle cell lines, C2C12 myoblasts were from American Type Culture Collection (cat#ATCC^®^ CRL1772^™^, ATCC, Manassas, USA). Cell culture equipment was from VWR international (VWR, Mississauga, Canada). Dulbecco's modified Eagle's medium (DMEM), protein-free T20 (TBS) blocking buffer and phosphate buffered saline (PBS) were obtained from ThermoFisher Scientific (Invitrogen, Waltham, USA). Fetal bovine serum (FBS) and horse serum (HS) were purchased from GE Healthcare Life Sciences (Hyclone, Utah, USA). Antibiotics were from Sigma-Aldrich (Oakville, Canada), and May-Grünwald and Giemsa Stain Solutions were from Wako Pure Chemical Industries (Toronto, Canada). Antibodies for western blot were all purchased from Santa Cruz Biotechnology (Texas, USA) except of MitoProfile Total OXPHOS from Abcam (Toronto, Canada). Sodium dodecyl sulfate polyacrylamide gel electrophoresis (SDS-PAGE), transfer buffer (Tris glycine buffer), washing buffer (Tris buffer saline) and western blot chemiluminescent solution (Clarity western ECL substrate solution) were from Bio-Rad Laboratories (Mississauga, Canada). Monoclonal anti-SPARC and rSPARC were obtained from ThermoFisher Scientific (R&D Systems, Ottawa, Canada).

### Myoblasts culture and ECM/mitochondrial protein expression levels measurement in proliferating C2C12 cells

C2C12 mouse adherent myoblasts were cultured in grown in DMEM supplemented with 10% FBS, 100 U/ml of penicillin, and 100 mg/ml of streptomycin (growth medium, GM) and they were maintained at 37°C under a 5% CO_2_ atmosphere [24]. One day before adding rSPARC or anti-SPARC, cells were trypsinezed and plated at density of 4×10^5^/well in 12-well plates (day 1). On day 2, cells were cultured in different conditions and kept in GM supplement with rSPARC and/or anti-SPARC for 48 h. On day 4, proteins extraction was performed as described below (Immunoblot analysis). Collagen 1a1 and fibronectin protein expression levels as well as the expression of five different mitochondrial OXPHOS proteins were measured.

### Myoblasts differentiation, ECM/mitochondrial and myogenin protein expression levels measurement

C2C12 myoblasts were trypsinized and plated in 12-well at 4×10^5^ cells/well with GM (day 0). 24 h after plating, cells reached 80–90% confluence and the confluent C2C12 myoblasts were induced to differentiate by being transferred into DMEM with 2% heat-inactivated horse serum and 100 U/ml of penicillin, and 100 mg/ml of streptomycin (differentiation medium, DM) for 5 days. Thereafter medium was changed every other day. On day 6, proteins extraction was performed and myogenin, collagen 1a1, fibronectin and mitochondrial OXPHOS protein levels were measured by western blot.

### Measurement of collagen 1a1 expression after induction/inhibition of SPARC in the mature myotubes

The differentiation of C2C12 myoblasts was performed for 5 days without any exogenous induction and/or inhibition of SPARC. On day 6, mature myotubes are generally fully formed, rSPARC and anti-SPARC were added to the DM and kept for 2 days. On day 8, proteins extraction followed by western blot were performed to analyze collagen 1a1 protein level after induction/inhibition of SPARC in mature myotubes.

### Exogenous SPARC inhibition/induction

Monoclonal anti-SPARC (Mouse SPARC MAb "Clone 124413", Rat IgG2B, R&D systems, catalogue number: MAB942) (10–40 μg/ml) and rSPARC (R&D systems, catalogue number: 942-SP-050) (2–8 μg/ml) were directly added into GM or DM. For our fourth experimental condition, first, anti-SPARC was added into GM or DM, and then rSPARC was appended at 1 h later.

### Measurement of cell fusion

To demonstrate the implication of SPARC in the differentiation of mononucluated myoblasts, a simple effective quantitative method was used to quantify myotube formation using May-Grünwald and Giemsa staining since myotubes are darkly stained, which makes it easy to distinguish nuclei and myotubes [25, 26]. Only slight modifications were brought. C2C12 cells were grown in GM and seeded at 4×10^4^ cells/well in 24-well plates (day 0). On day 1, the differentiation was induced in DM with four different experimental conditions (day 1) for 5 days. DM with all experimental conditions was changed each 48 h. On day 6, cultured cells were fixed for 5 min in methanol, dried for 10 min and incubated for 5 min in May-Grünwald diluted solution (1:3 in sodium phosphate buffer). Cells were washed twice with distilled water (DW), stained with Giemsa diluted solution (1:10 in DW) for 20 min, and then rinsed 3× with DW. All the preparations were observed under light microscopy (Zeiss Axiophot, Germany) and microscopic images were captured at 30× and 100× of magnification. Cells were considered to be fused only if at least three nuclei were present in each myotube. Each value represents the average of at least 10 randomly selected fields. The fusion index was calculated from the ratio of nuclei number in the myotubes versus the total number of nuclei.

### lmmunoblot analysis

After the proliferation as well as the differentiation of C2C12 myoblasts, the resulting culture were washed twice with PBS and scraped on ice by using cell lifter. Then, the PBS containing cells were centrifuged at 3000 rpm for 4 min at 4°C. The pellets were resuspended in radio-immunoprecipitation assay (RIPA) buffer supplemented with protease inhibitors cocktail and incubated on ice for 15 min. Lysates were sonicated 3× for 1 sec, centrifuged at 14,000 rpm for 5 min at 4 °C. The whole cell extracts were stored at -80 °C until use. Pooled samples were made and used to decide the amount of the protein to load for each antibody. This will assure that the densitometric data for each target protein will be within the linear dynamic (quantitative) range to give accurate and reproducible results reflecting the true biology between samples in the study set [27]. The most popular loading controls include housekeeping such as beta-actin, tubulin and GAPDH, however these proteins are generally highly expressed in samples and are overloaded in the gel lane with the target protein such that they would not serve to normalize the loading [27]. Thus, we loaded the same pooled samples in each gel to be used as a control to normalize the difference between each membrane. Five to thirty μg of proteins from each cell lysate including pooled samples were separated by electrophoresis through SDS-PAGE and transferred onto polyvinylidene fluoride (PVDF) membrane. Once the transfer was completed, membranes were incubate with diluted 10× RedAlert for 5 min following by washing with DW as previously described [28] and pictures were taken to normalize the quantity of total protein loaded and the differences between the membranes using the pooled samples or control for mitochondrial proteins (see S1 Fig for RedAlert pictures). The ratio of samples/pooled samples from RedAlert picture (RM) was calculated for each membrane and used to normalize the quantity of total protein loaded. Then, membranes were blocked using suitable blocking buffer and incubated with primary antibodies (see S1 Table for western blot conditions). After washed (3×) with appropriates washing buffers, membranes were incubated with species-appropriate horseradish peroxidase (HRP)-conjugated secondary antibodies, washed (3×) again and finally the visualization of the immune complexes was carried out with an enhanced chemiluminescent reagent. The intensity of bands was measured using Image J software. The density of each lane on the on the film (DF) was expressed as a ratio to each pooled sample on the same film to normalize the difference of each membrane. Then, the quantity of protein loaded was normalized by dividing DF by RM as previously described [29].

### Statistical analysis

The data presented are repeated measures from three independent experiments (three different passages of C2C12 cells to measure the effect of SPARC on C2C12 phenotype, not only on one passage of this cell line), except for the myoblasts fusion data. One-way ANOVA with repeated measurements was used to adjust the validations of the different passages and a contrast analysis was performed. P value was set at < 0.05 after the Bonfferoni adjustments. For the myoblasts fusion data, the same passage was used with three repetitions for each condition, thus, one-way ANOVA followed by the Tukey's HSD post-hoc test was used. All results are reported as means ± SEM.

## Results

### Effect of SPARC on the modulation of structural matrix proteins during the proliferation of C2C12 myoblasts

**Fig 1:**
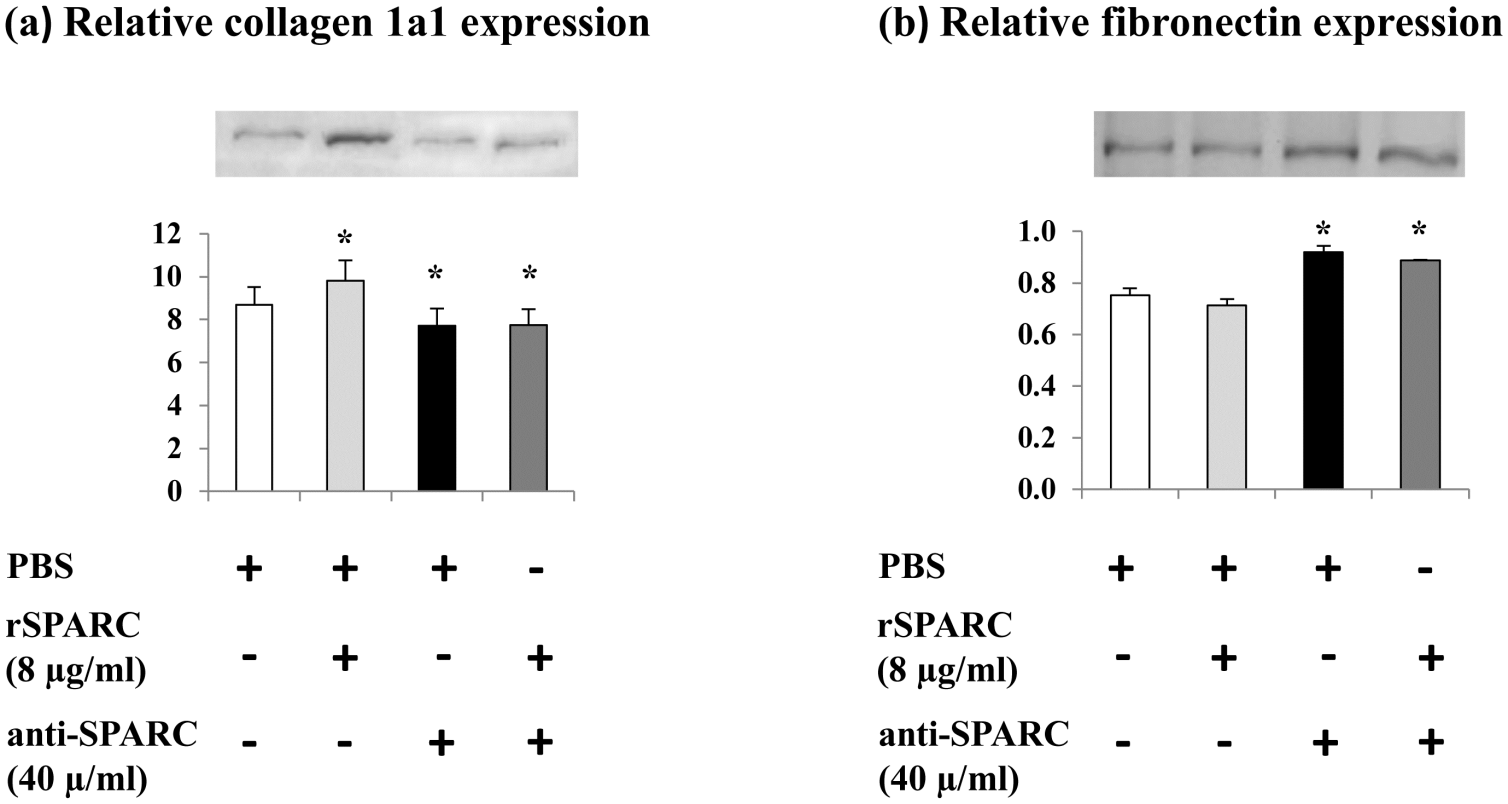
Effect of SPARC on the modulation of ECM proteins in proliferating C2C12 cells. Representative western blots for collagen 1a1 and fibronectin expressions in C2C12 myoblasts after induction/inhibition of SPARC. C2C12 myoblasts were cultured in GM. One day before adding anti-SPARC (40 μg/ml) or rSPARC (8 μg/ml), cells were trypsinezed and plated at density of 4×10^5^/well in 12-well plates. Cells were cultured in four different conditions for 48 h. Proteins extraction was performed as described above and collagen 1a1 and fibronectin protein expression levels were measured by western blot. The experiments were repeated three times (three different passages of C2C12 cells) and a representative western blot image was shown. Data were expressed as a ratio to the positive control (pooled samples). One-way ANOVA with repeated measurements was used to adjust the validations of the different passages and a contrast analysis was performed. P value was set at < 0.05 after the Bonfferoni adjustments. (a) Thirty μg of whole cell lysate proteins were loaded to measure collagen 1a1 levels in C2C12 proliferating myoblasts. Addition of SPARC induced collagen 1a1 expression, however, SPARC inhibition decreased it. (b) Five μg of the proteins from proliferating cells were loaded to measure fibronectin levels. Fibronectin expression trended to decrease after rSPARC addition and anti-SPARC increased it. Abbreviations: PBS: phosphate buffered saline, rSPARC: recombinant SPARC protein, and anti-SPARC: anti-SPARC antibody. *Significant differences between experimental conditions.

SPARC is reported to bind fibrillar collagens I [30] on its binding [31]. In SPARC-null cells, decreased collagen type I expression [32] and enhanced collagen assembly with SPARC overexpression [33] are observed. Moreover, SPARC is “anti-adhesive” protein and induces focal adhesion disassembly [16]. On the other hand, fibronectin, ECM adhesive glycoprotein, is found to be expressed in all stages of primary myogenesis [34] and may play an important role in muscle development [35]. Thus, to study the implication of SPARC in ECM remodeling, we examined the effect of SPARC on collagen 1a1 and fibronectin expression during the proliferation of C2C12 cells. Our results showed that exogenous SPARC induction (8 μg rSPARC/ml) increased the expression level of collagen 1a1 and trended to decrease fibronectin expression (p < 0.1) in the proliferating C2C12 myoblasts. However, SPARC inhibition (40 μg anti-SPARC/ml) decreased collagen 1a1 expression and increased fibronectin expression (Fig 1). These results provide evidence that SPARC modulates ECM proteins during myoblasts proliferation.

### Effect of SPARC on the modulation of OXPHOS proteins in proliferating myoblasts

**Fig 2:**
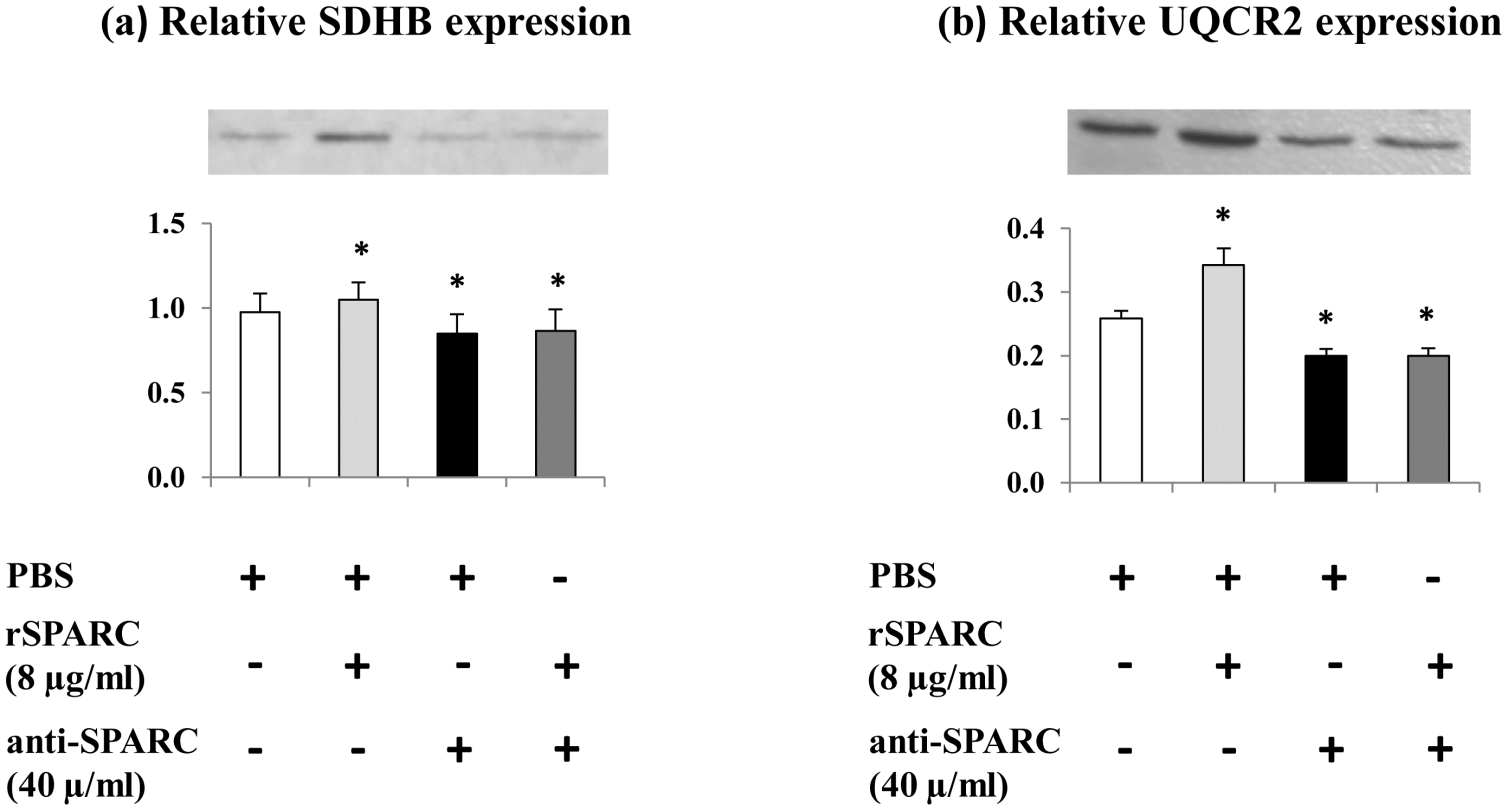
Modulation of mitochondrial proteins expression after exogenous induction/inhibition of SPARC in proliferating myoblasts. C2C12 cells were cultured in four different conditions which were kept in GM supplement with/without rSPARC and/or anti-SPARC for 48 h. Extracted cells lysates were prepared to perform western blot and the expression of mitochondrial OXPHOS proteins were measured using 30 μg of whole cell lysate proteins. The experiments were repeated three times (three different passages of C2C12 cells) and a representative western blot image was shown. Data were expressed as a ratio to the positive control (pooled samples). One-way ANOVA with repeated measurements was used to adjust the validations of the different passages and a contrast analysis was performed. P value was set at < 0.05 after the Bonfferoni adjustments. The addition of rSPARC (8 μg/ml) increased SDHB (a) and UQCR2 (b) proteins levels, whereas, anti-SPARC (40 μg/ml) decreased it. Abbreviations: SDHB: succinate dehydrogenase iron-sulfur subunit beta, UQCR2: ubiquinol-cytochrome c reductase core protein 2, PBS: phosphate buffered saline, rSPARC: recombinant SPARC protein, and anti-SPARC: anti-SPARC antibody. *Significant differences between experimental conditions.

Mitochondrial dysfunction is found to be implicated in the development of many diseases in the skeletal muscle [36, 37]. There is increased evidence that mitochondria may function as detectors for changes in ECM composition and changes in mitochondrial functioning modify the ECM [38, 39]. Furthermore, SPARC which modulated ECM proteins (Fig 1) interacts with AMPK [21] which is known to induce the master regulator of mitochondrial biogenesis [22]. Thus, to examine the effect of exogenous induction/inhibition of SPARC on mitochondrial proteins expression, same cell culture conditions and procedures were followed as above and the expression of mitochondrial OXPHOS proteins were measured using the total OXPHOS antibody Cocktail (1:200). Our data demonstrated that the addition of rSPARC (8 μg/ml) in muscle cell culture medium increased the expression of two mitochondrial proteins (succinate dehydrogenase assembly factor 4 (SDHB) and ubiquinol-cytochrome-C reductase complex core protein 2 (UQCR2)). In contrast, the addition of anti-SPARC (40 μg/ml) decreased the content in these key oxidative energy components (Fig 2). These findings are the first proof that SPARC can modulate expression of mitochondrial proteins during myoblasts proliferation.

### Involvement of SPARC in the differentiation of C2C12 myoblasts

C2C12 *in vitro* cell culture is a great model to study the muscle development and differentiation because these cells have all the characteristics needed for the study of myogenesis: differentiates rapidly, forms contractile skeletal myotubes and produces characteristic muscle proteins [40].

**Fig 3:**
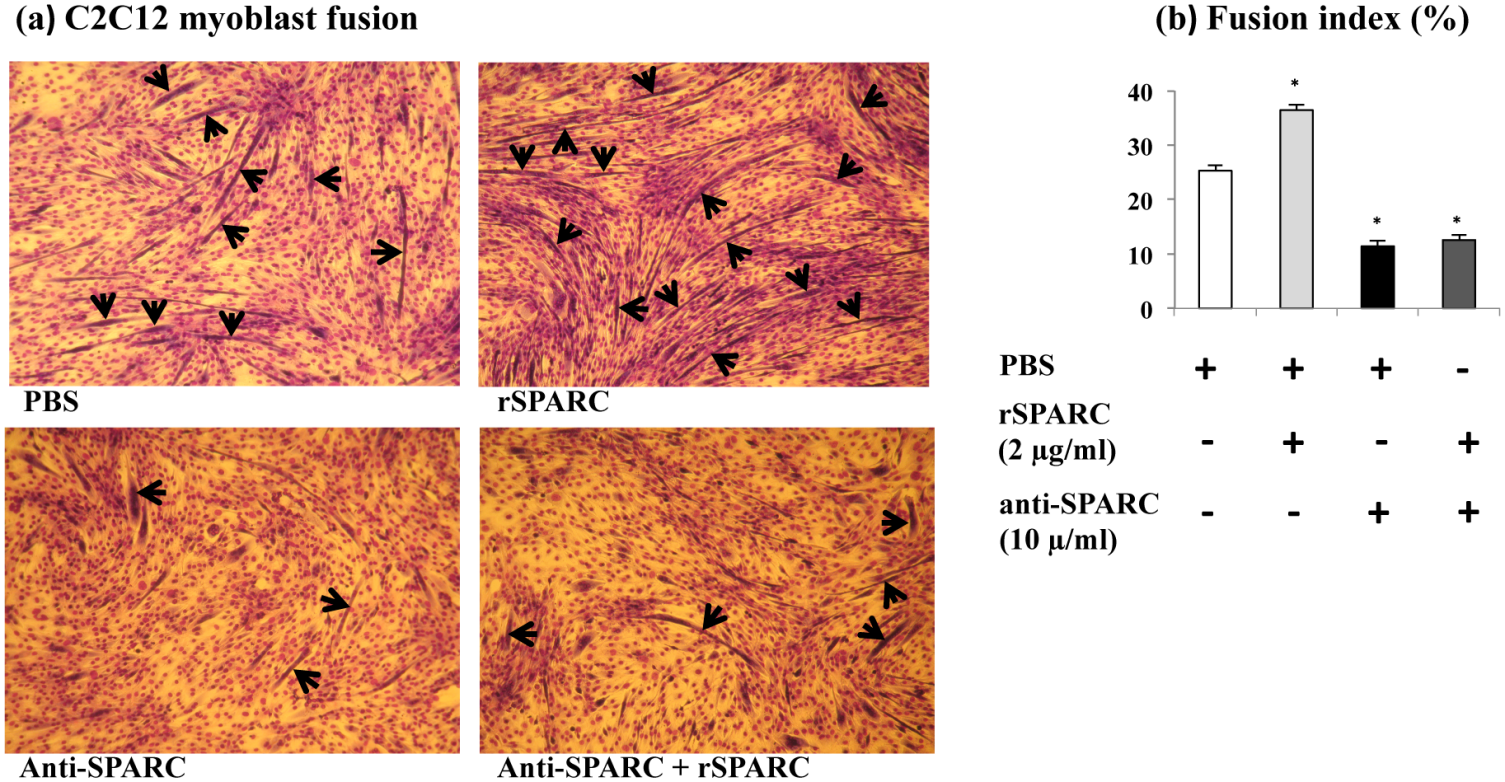
Involvement of SPARC in myoblasts fusion. Myoblasts (4×10^4^ cells/well in 24-well plates) were induced to differentiate in DM with/without the induction/inhibition of SPARC for 5 days. The mediums were changed each 48 h. C2C12 were stained with May-Grünwald and Giemsa solutions, and the stained wells were observed under light microscopy. One-way ANOVA followed by the Tukey's HSD post-hoc test was used. All results are reported as means ± SEM. The addition of rSPARC (2 μg/ml) induced myotube formation, whereas, anti-SPARC (10 μg/ml) inhibited it. (a) Captured microscopic images of C2C12 fusion. The images were captured using 30× objectives under light microscopy (see S2 Fig for 100× objectives). Examples of multinucleated myotubes were shown by arrows. (b) Fusion index was calculated from 10 different photos per condition. Abbreviations: PBS: phosphate buffered saline, rSPARC: recombinant SPARC protein, and anti-SPARC: anti-SPARC antibody. *Significant differences between experimental conditions (n = 3): p < 0.05.

It has been demonstrated that ECM is involved in the control of muscle differentiation [41]. The expression of SPARC is detected both in satellite cells/myoblasts and myotubes and muscle fibers, suggesting a crucial role for SPARC in the skeletal muscle compartment [12]. To demonstrate the implication of SPARC in the differentiation of mononucluated myoblasts, a simple effective method was employed to quantify myotubes formation [25, 26]. The differentiation was induced in DM with four different experimental conditions for 5 days. The DM with/without exogenous induction (2 μg rSPARC/ml) and/or inhibition (10 μg anti-SPARC/ml) of SPARC were changed each 48 h. C2C12 were stained with May-Grünwald and Giemsa solutions and observed under light microscope. As expected, the exogenous induction of SPARC increased the fusion of myotubes in the differentiating C2C12 cells, whereas SPARC inhibition decreased the muscle cell fusion (Fig 3). These results confirm that anti-SPARC prevents the differentiation of C2C12 myoblasts [23] and provides new evidence that SPARC induces differentiation of C2C12 cells.

**Fig 4:**
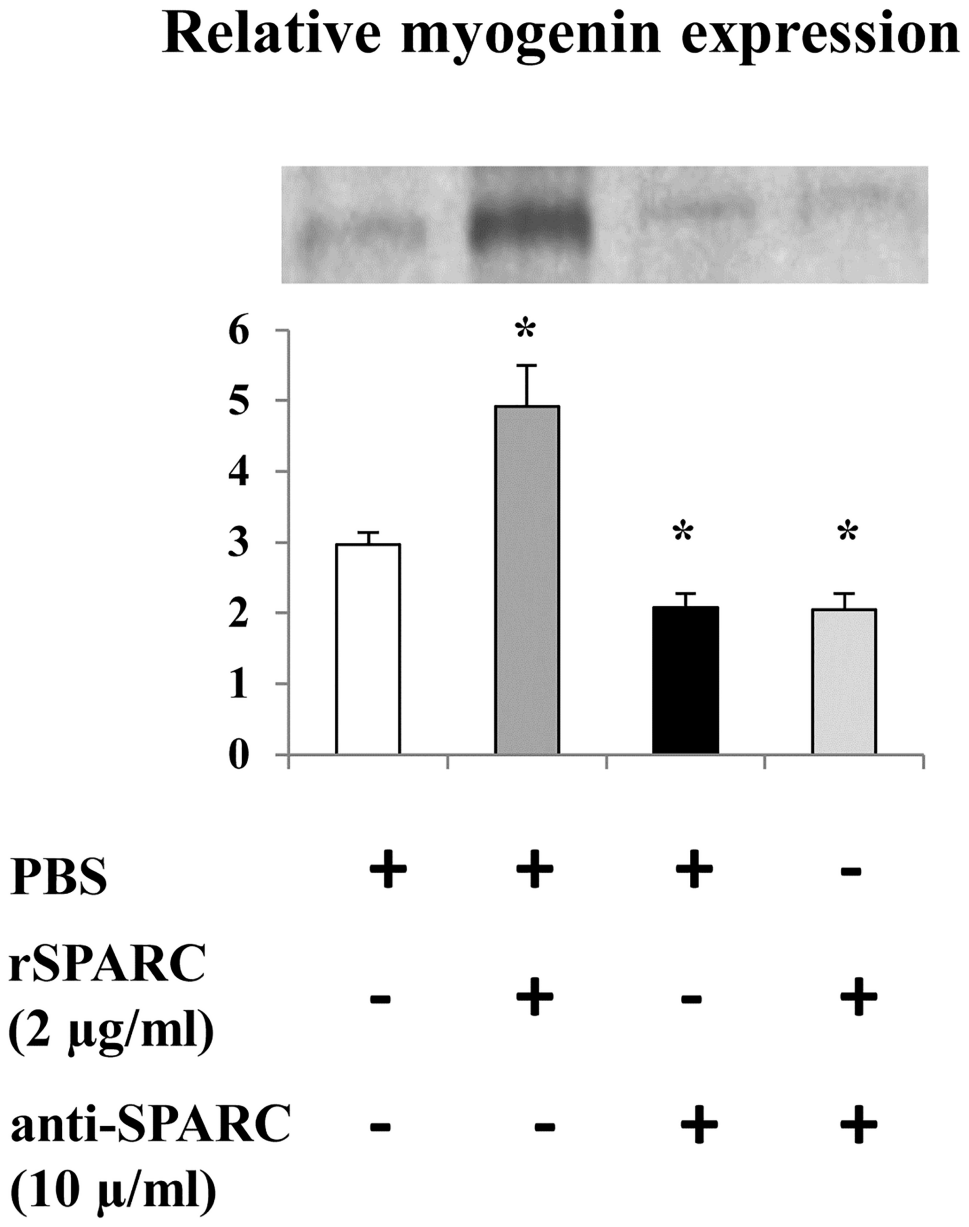
Exogenous effect of SPARC on myogenin expression in differentiating myoblasts. Confluent C2C12 myoblasts were trypsinized and plated in 12-well at 4×10^5^ cells/well with GM. The myogenic differentiation was induced in DM with/without the induction/inhibition of SPARC for 5 days, and the mediums were replaced every 2 days. Proteins extraction was performed and five μg from total cell extracts were analyzed by western blot using anti-myogenin antibody. The experiments were repeated three times (three different passages of C2C12 cells) and a representative western blot image was shown. Data were expressed as a ratio to the positive control (pooled samples). One-way ANOVA with repeated measurements was used to adjust the validations of the different passages and a contrast analysis was performed. P value was set at < 0.05 after the Bonfferoni adjustments. The SPARC induction increased myogenin expression and SPARC inhibition decreased it. Abbreviations: PBS: phosphate buffered saline, rSPARC: recombinant SPARC protein, and anti-SPARC: anti-SPARC antibody. *Significant differences between experimental conditions.

Myogenin is an important regulator of skeletal muscle cell differentiation [42, 43]. To confirm the involvement of SPARC in the differentiation of muscle cells, myogenin expression level was measured by western blot after the induction (2 μg rSPARC/ml)/inhibition (10 μg anti-SPARC/ml) of SPARC during 5 days of C2C12 myoblasts differentiation. Our data showed that during the differentiation of C2C12 cells, SPARC induction increased, whereas the inhibition decreased myogenin expression, respectively (Fig 4). This data demonstrates a key role of SPARC in the differentiation of muscle cells.

### SPARC as a modulator of structural matrix proteins during the differentiation of muscle cells

**Fig 5:**
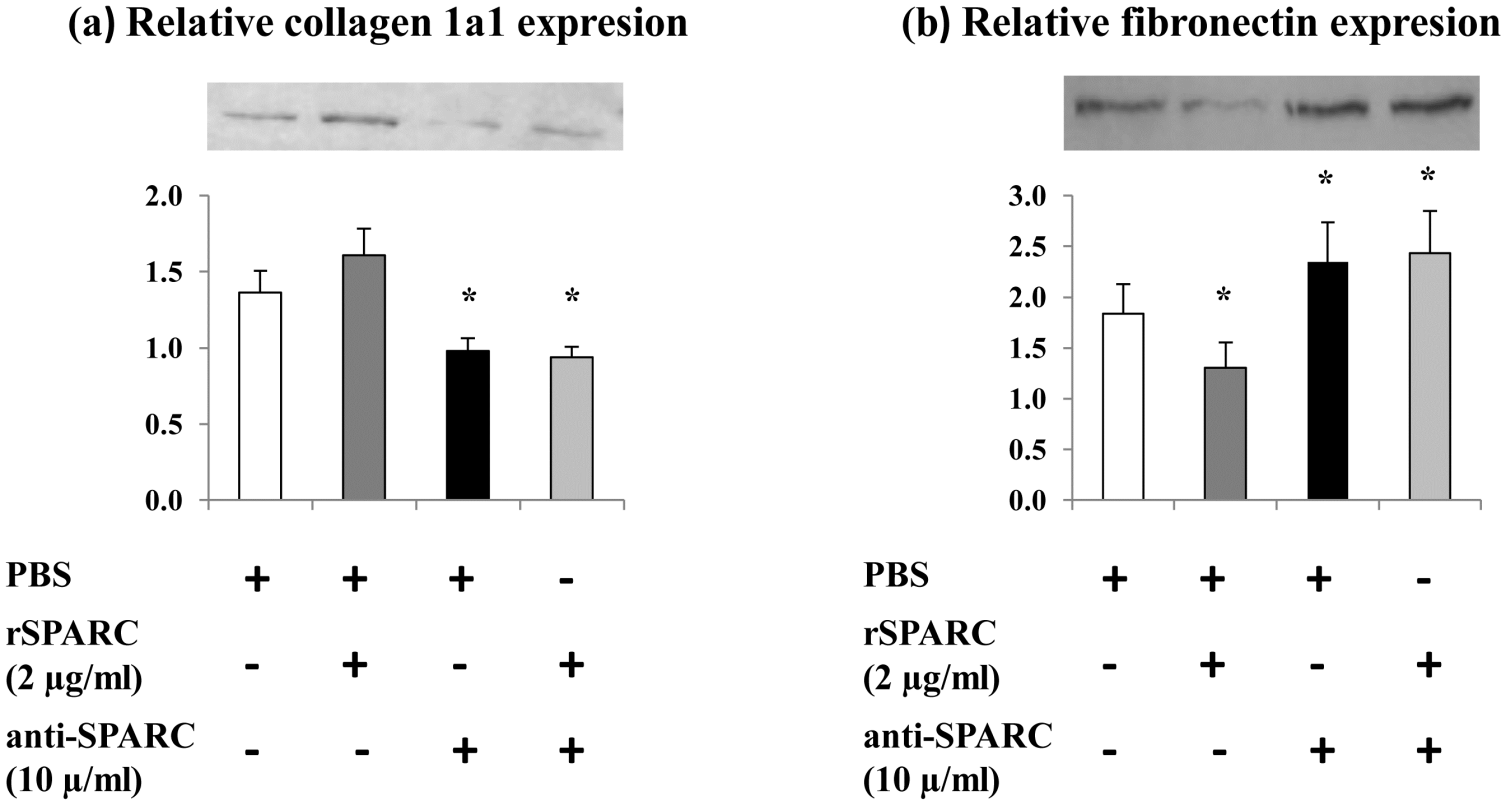
SPARC effect on ECM proteins in differentiating myoblasts. C2C12 cells were cultured in DM for 5 days with/without exogenous rSPARC (2 μg/ml) and/or anti-SPARC (10 μg/ml). The mediums were changed each 2 days. Proteins extraction was performed, and western blot analysis was done on RIPA soluble lysates to indicate proteins. The experiments were repeated three times (three different passages of C2C12 cells) and a representative western blot image was shown. Data were expressed as a ratio to the positive control (pooled samples). One-way ANOVA with repeated measurements was used to adjust the validations of the different passages and a contrast analysis was performed. P value was set at < 0.05 after the Bonfferoni adjustments. (a): collagen 1a1 levels were measured using the 30 μg proteins and the results showed that the induction of SPARC increased collagen 1a1 expression, however, an opposite effect of anti-SPARC was observed. (b) Five μg of the proteins were loaded to measure fibronectin levels. A decrease of fibronectin expression with rSPARC and an increase after anti-SPARC were observed. Abbreviations: PBS: phosphate buffered saline, rSPARC: recombinant SPARC protein, and anti-SPARC: anti-SPARC antibody. *Significant differences between experimental conditions.

SPARC specifically binds several ECM proteins including collagen and is implicated in the regulation of cell-matrix interactions [44]. SPARC also modulates the production of ECM molecules [45]. Further, *In vitro* studies have been demonstrated that the inhibition of collagen suppresses the differentiation of myoblasts *in vitro*, suggesting that collagen is necessary for myogenesis [46, 47]. Thus, collagen 1a1 and fibronectin protein levels were measured to study the effect of SPARC on the modulation of the ECM in differentiating C2C12 cells. After the induction of C2C12 differentiation for 5 days with/without exogenous induction/inhibition of SPARC, whole cell lysates were subjected to western blot using anti-collagen 1a1 and anti-fibronectin antibodies. The addition of rSPARC (2 μg/ml) trended to increase the expression levels of collagen 1a1 (p < 0.1) and decreased fibronectin in the differentiating C2C12 cells. SPARC inhibition (10 μg anti-SPARC/ml) decreases collagen 1a1 and increases fibronectin (Fig 5). The obtained results suggested that SPARC acts as a modulator of muscle ECM during the differentiation.

### Effect of SPARC on the expression of mitochondrial OXPHOS proteins in the differentiating C2C12 cells

**Fig 6:**
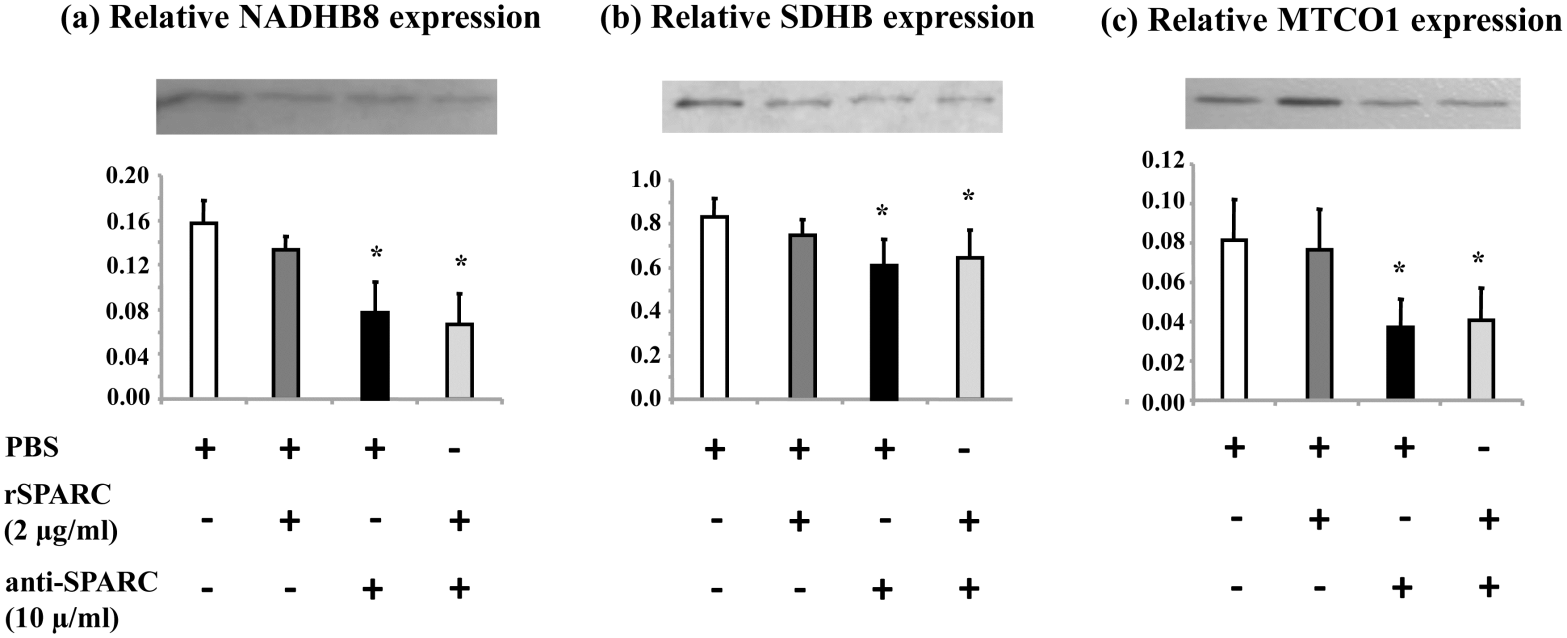
Modulation of mitochondrial OXPHOS proteins expression after exogenous inhibition of SPARC in differentiating myobalsts. The myogenic differentiation was induced in DM supplemented with the induction/inhibition of SPARC for 5 days. DM with rSPARC (2 μg/ml) and anti-SPARC (10 μg/ml) was replaced each 2 days. Proteins extraction was performed, and extracted cells were prepared to perform western blot and the expression of mitochondrial OXPHOS proteins were measured using 30 μg of whole cell lysate proteins. The experiments were repeated three times (three different passages of C2C12 cells) and a representative western blot image was shown. Data were expressed as a ratio to the positive control (pooled samples). One-way ANOVA with repeated measurements was used to adjust the validations of the different passages and a contrast analysis was performed. P value was set at < 0.05 after the Bonfferoni adjustments. The expression of three different mitochondrial proteins was shown to be reduced after SPARC inhibition. Abbreviations: SDHB: succinate dehydrogenase iron-sulfur subunit beta, NADHB8: NADH dehydrogenase ubiquinone 1 beta subcomplex subunit 8, MTCO1: cytochrome c oxidase 1, PBS: phosphate buffered saline, rSPARC: recombinant SPARC protein, and anti-SPARC: anti-SPARC antibody. *Significant differences between experimental conditions.

It is well known that mitochondria are potential regulator of myogenesis and skeletal muscle cells devoid of mitochondria cannot differentiate [48]. The presence of ECM is also essential for the formation of myotubes [49]. In the Figs 3 and 4, we demonstrated that SPARC induced myotubes formation and modulated ECM proteins expression during the differentiation of C2C12 cells. Thus, to study the exogenous effect of SPARC on mitochondrial proteins expression during the differentiation of C2C12 cells, mononucleated myoblasts were treated with rSPARC and/or anti-SPARC during 5 days of myoblasts differentiation. The mitochondrial OXPHOS proteins level was measured by western blot. The results showed that only exogenous SPARC inhibition decreased the expression of three mitochondrial proteins (NADH dehydrogenase (ubiquinone) 1 beta sub-complex 8 (NDUFB8), SDHB and cytochrome c oxidase I (MTCO1)) during the differentiation of C2C12 cells (Fig 6). According to this result, a modulator role of SPARC on mitochondrial proteins expression during muscle cells differentiation is suggested.

### Modulation of collagen 1a1 expression after induction/inhibition of SPARC in the mature myotubes

**Fig 7:**
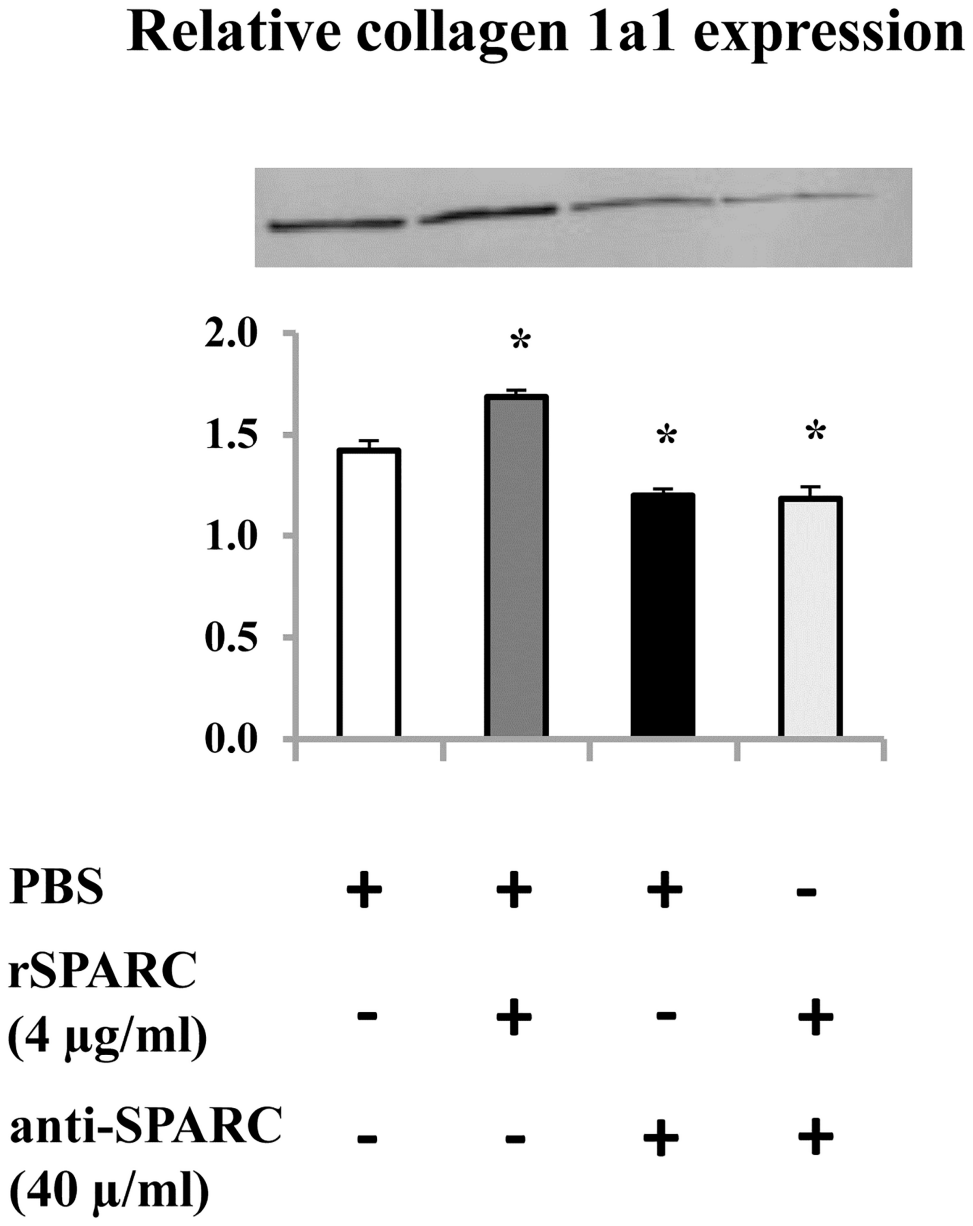
Modulation of collagen 1a1 expression after induction/inhibition of SPARC in the mature myotubes. C2C12 myoblasts were cultured in DM for 5 days. On day 6, exogenous rSPARC (4 μg/ml) and anti-SPARC (40 μg/ml) were added and kept for 48 h. Proteins extraction was performed on day 8. Total cell extracts (30 μg proteins per lane) were subjected to western blot analysis with anti-collagen 1a1 antibody. The experiments were repeated three times (three different passages of C2C12 cells) and a representative western blot image was shown. Data were expressed as a ratio to the positive control (pooled samples). One-way ANOVA with repeated measurements was used to adjust the validations of the different passages and a contrast analysis was performed. P value was set at < 0.05 after the Bonfferoni adjustments. Abbreviations: PBS: phosphate buffered saline, rSPARC: recombinant SPARC protein, and anti-SPARC: anti-SPARC antibody. *Significant differences between experimental conditions.

It has been shown that the expression and the organization of collagen are critical to both skeletal muscle function and development [50]. Moreover, the production of collagen I is a requisite for the association of SPARC with ECM [31]. Thus, we studied the effect of exogenous SPARC on collagen 1a1 expression in myotubes. The differentiation of C2C12 myoblasts were performed for 5 days without any exogenous induction and/or inhibition of SPARC. On day 6, mature myotubes are generally formed, rSPARC and anti-SPARC were added to the DM and kept for 2 days. On day 8, proteins extraction followed by western blot was performed to analyze collagen 1a1 protein level after induction/inhibition of SPARC in mature myotubes. SPARC induction increased collagen 1a1 expression in C2C12 myotubes, whereas a decrease was observed by SPARC inhibition (Fig 7). The obtained result confirmed the modulator effect of SPARC on ECM proteins expression in the differentiated muscle cells as seen in proliferating and differentiating C2C12 myoblasts.

## Discussion

In this study, we investigated the modulator effect of SPARC on the ECM and mitochondrial proteins expression in muscle cells. Moreover, we demonstrated the involvement of SPARC in the differentiation of C2C12 myoblasts.

### SPARC as a modulator of the ECM in muscle cells

ECM is a crucial multifunctional dynamic structure which modulates different cellular functions. It is well known that skeletal muscle cells interact with the ECM resulting in the mediation of their morphogenesis [2]. Previous studies have demonstrated the requirement for the ECM and its components in the differentiation of myoblasts [43, 51]. Furthermore, it has been reported that collagen is the principal structural protein in ECM skeletal muscle [52] and an early data has highlighted the importance of collagen 1 in muscle development [53]. SPARC is a collagen-binding matricellular glycoprotein secreted into the ECM and may have a fundamental role in muscle compartment [12]. The importance of the production of collagen 1 in the association between SPARC and the ECM as well as the interactions of SPARC with the collagen I precursor and procollagen 1 have been demonstrated [31]. The mediator effect of SPARC on the association of procollagen I with cells, its processing and incorporation into the ECM has been shown in dermal fibroblasts [54]. Moreover, the reduction of collagen 1 content in SPARC-null mice [20] and the deterioration of SPARC deposition in type 1 collagen deficient mice [18] have been elucidated. Taken all these studies, a functional relationship between SPARC and collagen 1 is evident. However, to our knowledge, no study has shown the effect of SPARC on collagen 1 protein expression during the myogenic process. Thus, we described here that exogenous induction of SPARC increased collagen 1 protein expression during the proliferation and after the formation of mature myotubes and trended to increase collagen 1 protein level during the differentiation. On the other hand, SPARC inhibition decreased collagen 1 expression during the three steps of the myogenesis process. One possibility to explain the effect of SPARC on collagen 1 protein expression during the myogenic process is their direct binding [31]. Importantly, our data supports the findings of Francki et al. in kidney mesangial cells where SPARC-null cells display diminished expression of collagen type I mRNA and protein [32]. The same study has reported that the addition of rSPARC to SPARC-null cells restores the expression of collagen type I mRNA [32].

Fibronectin is a multifunctional ECM glycoprotein that plays a structural role in the ECM and binds several ECM molecules [55]. Fibronectin belongs to the components of cell-ECM adhesion complexes and its interaction with integrin receptors mediates cell attachment, migration and signaling [56]. In myoblasts, it has been demonstrated that fibronectin promotes cell attachment for important physiological processes such as proliferation and differentiation [57]. The same study has reported the influence effect of the conformational changes in fibronectin on C2C12 muscle cells [57]. In contrast, SPARC has a counter-adhesive activity by the disruption of cell-ECM interactions [58], although, this activity of SPARC depends on the target cell type and the origin of SPARC protein. The mechanism governed the dissociation of cell adhesion after SPARC addition is not completely clear [16]. Furthermore, in vascular smooth muscle cells, SPARC inhibits cell proliferation and cell cycle progression [15, 59], while fibronectin promotes cell proliferation [60]. Moreover, cell adhesion has been described as a calcium-binding mechanism [61]. Indeed, SPARC is a calcium-binding protein [62] and an interaction of calcium with fibronectin has been also elucidated [63].

Here, we showed that exogenous SPARC induction trended to decrease fibronectin expression in the proliferating C2C12 myoblasts and decreased fibronectin in the differentiating C2C12 cells. However, SPARC inhibition increased fibronectin expression in proliferating and differentiating C2C12 cells. Hence, SPARC can be implicated in the ECM remodeling through the modulation of structural matrix proteins expression in muscle cells.

### Involvement of SPARC in the differentiation of C2C12 myoblasts

The organization and the composition of ECM are influenced by exercise-induced changes, fiber maturation and myogenesis-related changes [64, 65]. SPARC is induced after exercise [10, 66] and during recovery from muscle injury [12]. Moreover, ECM components are involved in myoblasts differentiation. For instance, laminin, a structural matrix protein, enhances myotube formation, whereas fibronectin inhibits this process [67, 68]. An important study has demonstrated the implication of SPARC in the myogenesis of skeletal myoblasts in *vitro*. The authors have reported: 1) an increase of SPARC expression during mouse myoblasts differentiation, 2) SPARC expression may be controlled by calcium-dependent pathway in myogenesis, and 3) the treatment with anti-SPARC almost completely preventes myobalsts differentiations [23].

Therefore, we confirmed the effect of anti-SPARC on the myoblasts differentiation and we investigated the induction of myoblasts fusion after rSPARC addition. Moreover, myoblasts differentiation is governed by myogenin expression [42, 43]. Consequently, we showed here that SPARC induction increased, whereas the inhibition decreased myogenin expression, respectively. Therefore, SPARC is importantly implicated in muscle differentiation, and this process can be regulated in part by MRFs expression, including myogenin, and by ECM remodeling.

Based on the involvement of SPARC in the differentiation of skeletal muscle cells and its key role in this process, we found that the addition of SPARC enhanced myotube formation through, may be in part, by decreasing fibronectin protein level. Our results correlate with other data showing that the alteration or the degradation of fibronectin is required for myoblasts fusion and also high level of fibronectin reduces myotube formation and that decrease and/or loss of fibronectin during myoblast fusion is closely correlated with the fusion of myoblasts [69, 70]. In addition, study of Chen has confirmed that during myoblasts differentiation, a reduction of fibronectin concentration on myoblast surface is observed [71].

### Effect of SPARC on the mitochondrial function of muscle cells

It is well known that the main role of mitochondria is energy production. However, evidence is increasing that mitochondria plays also a crucial role in cell regulatory, signaling events and in the response of cells to various stimulus [72]. In addition, mitochondria generate adenosine triphosphate (ATP) via OXPHOS complexes and plays fundamental role in cell proliferation as well as calcium signaling [73]. Further, previous studies have shown that alterations in mitochondria can affect ECM remodeling and vice versa collagen VI deficiency affects mitochondrial function in skeletal muscle [38, 74]. In the support of mitochondria can influence ECM, He et al. have demonstrated that the suppression of mitochondrial complex I influences expressions of ECM molecules and its related proteins [75]. Accordingly, a subtle relationship between the ECM and the mitochondria may be suggested and supported our hypothesis about the possible link between ECM remodeling and mitochondria function. On the other hand, the involvement of AMPK, a key regulator of many metabolic process in skeletal muscle, in the regulation of glucose metabolism in skeletal muscle has been elucidated [76]. In skeletal muscle, AMPK is known to induce mitochondrial function via direct phosphorylation of peroxisome-proliferator-activated receptor gamma coactivator 1alpha (PGC-1alpha), a master regulator of mitochondrial biogenesis [22]. Moreover, *in vitro* study reported the interaction of SPARC with AMPK and suggested the implication of SPARC in glucose metabolism via AMPK activation [21]. Thus, we investigate the effect of SPARC on mitochondrial OXPHOS protein expression during the proliferation and differentiation of C2C12 myoblasts. Our results demonstrated that in proliferating C2C12, SPARC addition increased the expression of mitochondrial proteins. On the other hand, the addition of anti-SPARC decreased their content. In differentiating C2C12 cells, only exogenous SPARC inhibition decreased the expression of mitochondrial proteins. The involvement of SPARC in the modulation of these key oxidative energy components during myogenesis process provides another way whereby ECM may regulate mitochondrial function.

## Conclusions

In summary, SPARC is a secreted peptide which has the potential to act similarly to a hormone in order to control important functions, such as extracellular cell growth and proliferation, and energy metabolism. This study is the first to demonstrate the effect of SPARC on the ECM remodeling and mitochondrial proteins expression during myogenesis process. The investigation of SPARC addition on myotube fusion as well as on MRFs (myogenin) expression confirms the involvement of SPARC in myogenic process. The decrease/increase of SPARC expression with ageing/exercise, respectively, and its involvement in the possible link between ECM and mitochondria may allow to understand the role of ECM remodeling in muscle integrity and to give an overview of ECM communications and its influence on muscle mitochondrial function. Finally, the implication of SPARC in the possible link between ECM remodeling and mitochondrial function provides new insight into the biological functions of SPARC and the pathway ECM/mitochondria may serve as a potential target to understand metabolic disorders related to ECM and mitochondria dysfunction which is known to be the major factor contributing to ageing and sarcopenia in muscle.

## Supporting information

S1 Fig RedAlert staining during the proliferation (a, b) and differentiation (c, d), and after the differentiation (e). **SM**: size marker, **0**: Pooled samples, **1**: PBS, **2**: rSPARC, **3**: anti-SPARC, **4**: Anti-SPARC+rSPARC. Abbreviations: ECM: extracellular matrix, PBS: phosphate buffered saline, OXPHOS: oxidative phosphorylation, rSPARC: recombinant SPARC protein, and anti-SPARC: anti-SPARC antibody.
(PDF)

S2 Fig Captured microscopic images of C2C12 fusion: 100X magnification. Abbreviations: PBS: phosphate buffered saline, rSPARC: recombinant SPARC protein, and anti-SPARC: anti-SPARC antibody.
(PDF)

S1 Table Western blot conditions. Abbreviations: AB: antibody, CAPS: N-cyclohexyl-3-aminopropanesulfonic acid, h: hours, Cat#: catalog number, ON: overnight, OXPHOS: oxidative phosphorylation, SDS: sodium dodecyl sulfate polyacrylamide, V: voltage.
(PDF)
