# Supplementary figures and images for: Implication of SPARC in the modulation of the extracellular matrix and mitochondrial function in muscle cells
Source: PLoS One. 2018 Feb 8;13(2):e0192714. doi: 10.1371/journal.pone.0192714 (PMC5805355; doi:10.1371/journal.pone.0192714)

S1 Fig

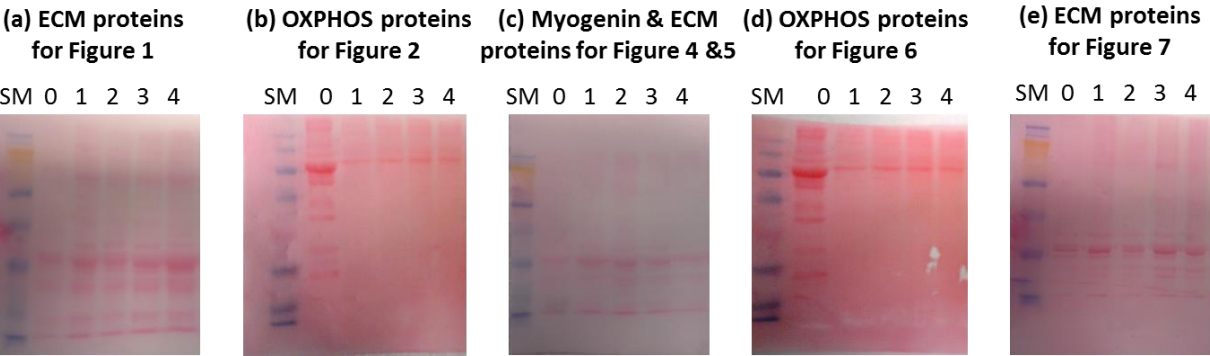

Supplement: S1 Fig — SM: size marker, 0: Pooled samples, 1: PBS, 2: rSPARC, 3: anti-SPARC, 4: Anti-SPARC+rSPARC. Abbreviations: ECM: extracellular matrix, PBS: phosphate buffered saline, OXPHOS: oxidative phosphorylation, rSPARC: recombinant SPARC protein, and anti-SPARC: anti-SPARC antibody. (PDF) [file pone.0192714.s001.pdf]

S2 Fig

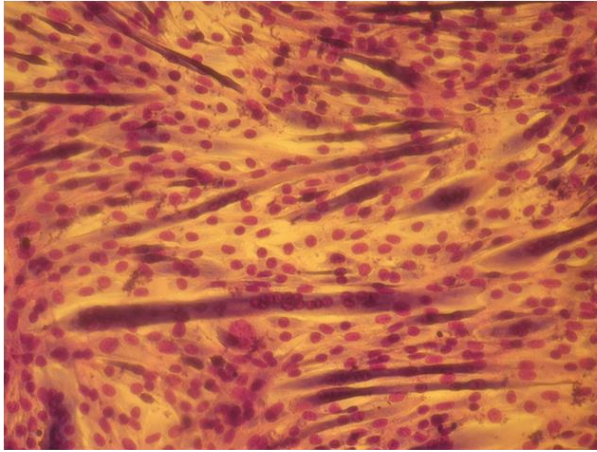

**PBS**

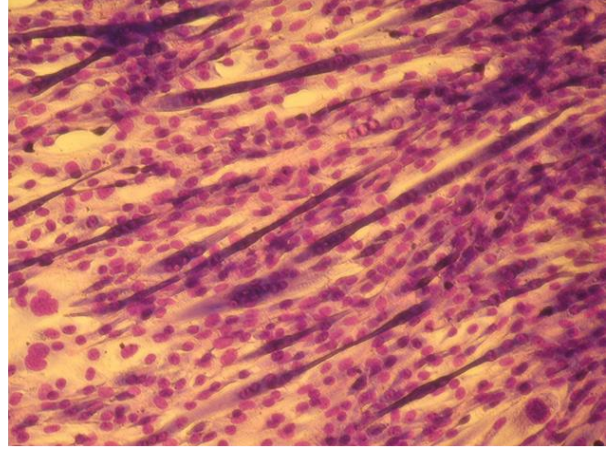

**rSPARC**

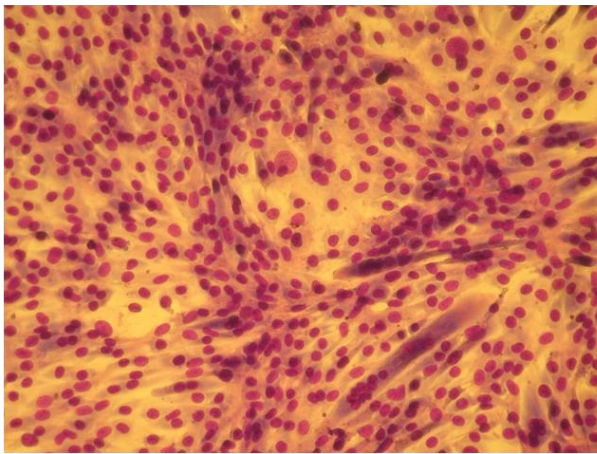

**Anti-SPARC**

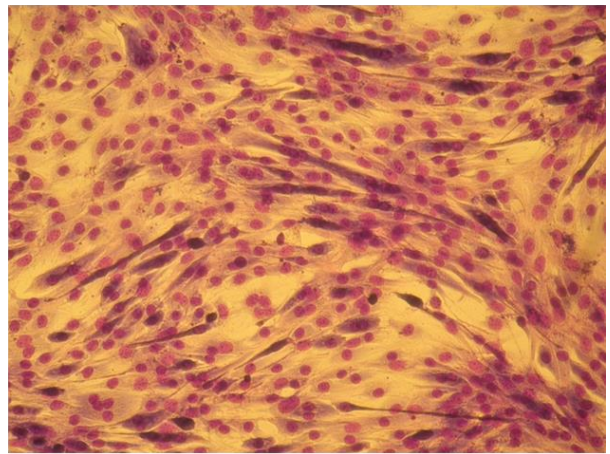

**Anti-SPARC + rSPARC**

Supplement: S2 Fig — Abbreviations: PBS: phosphate buffered saline, rSPARC: recombinant SPARC protein, and anti-SPARC: anti-SPARC antibody. (PDF) [file pone.0192714.s002.pdf]
