# Supplementary material for: Implication of SPARC in the modulation of the extracellular matrix and mitochondrial function in muscle cells
Source: PLoS One. 2018 Feb 8;13(2):e0192714. doi: 10.1371/journal.pone.0192714 (PMC5805355; doi:10.1371/journal.pone.0192714)
Supplement: S1 Table — Abbreviations: AB: antibody, CAPS: N-cyclohexyl-3-aminopropanesulfonic acid, h: hours, Cat#: catalog number, ON: overnight, OXPHOS: oxidative phosphorylation, SDS: sodium dodecyl sulfate polyacrylamide, V: voltage. (PDF) [file pone.0192714.s003.pdf]

S1 Table

Name	Protein (μg)	SDS gel (%)	Transfer (V)	Transfer (h)	Blocking (h)	1st AB (Cat#)	1st AB (Dilution)	1st AB (h)	2nd AB (Cat#)	2nd AB (h)	Note
**Collagen 1a1**	30	7	90	5	1	sc-8784-R	(1/200)	ON	sc-2004	1	-
**Fibronectin**	5	7	90	5	1	sc-8422	(1/800)	ON	sc-2005	1	-
**Myogenin**	5	12	75	2	1	sc-12732	(1/400)	ON	sc-2005	1	-
**Mitochondria (OXPHOS)**	30	12	75	3	ON	ab-110413	(1/200)	2	88-8887-31	2	Transfer buffer is 1X CAPS
